# Lipoprotein(a) testing trends in young ischemic stroke patients from 2015–2024: An analysis of 188,000 individuals

**DOI:** 10.1016/j.jstrokecerebrovasdis.2025.108513

**Published:** 2025-12-01

**Authors:** Mustafa Naguib, Brett C. Meyer, Francesca Felipe, Raphael E. Cuomo, Michael Wilkinson, Ehtisham Mahmud, Pam Taub, Harpreet S. Bhatia, Mattheus Ramsis

**Affiliations:** a Division of Cardiovascular Medicine, Department of Medicine, University of California San Diego, La Jolla, CA, USA; b Division of Vascular Neurology, Department of Neurosciences, University of California San Diego, La Jolla, CA, USA; c Department of Anesthesiology, School of Medicine, University of California San Diego, La Jolla, CA, USA

**Keywords:** Lipoprotein(a), Epidemiology, Ischemic stroke in young adults, Atherosclerosis, Stroke prevention, Bioinformatics, Large dataset, Electronic health record

## Abstract

**Background:**

Lipoprotein(a) [Lp(a)] is a genetically determined risk factor for myocardial infarction and stroke. Elevated Lp(a) >50 mg/dL (>125 nmol/L) is common and present in about 1 in 5 individuals. Although Lp(a) may be a cause of young ischemic stroke (age ≤60), limited data on national testing trends in this population are available, testing in the general population remains low overall, and different organizations have varying guidelines for testing. By determining the degree to which this population is tested, information on national testing trends of Lp(a) in young ischemic stroke patients may influence future guideline recommendations to increase Lp(a) testing. This study aims to use a large, real-world dataset to assess trends of Lp(a) testing in young ischemic stroke patients in the United States from 2015–2024.

**Methods:**

We performed a retrospective analysis of Lp(a) testing in young ischemic stroke patients across the United States from January 1, 2015 to December 31, 2024 using Epic Cosmos, a nationwide, de-identified electronic health record (EHR) dataset comprising over 300 million patient records from over 1,715 hospitals and 41,000 clinics, including from all 50 states, Washington D.C., Lebanon, and Saudi Arabia. The current count values for patients, hospitals, and clinics are available on the Epic Cosmos website. Although the Epic Cosmos data dictionary includes Lebanon and Saudi Arabia as standardized site locations, no patients from these countries were present in our analytic cohort; thus, all analyses were restricted to individuals within the United States. We evaluated the number of young ischemic stroke patients, defined as age ≤60 with history of an ischemic cerebrovascular accident (CVA), who had ever undergone Lp(a) testing, the testing rate per annual young ischemic stroke patients, geographical variation, and percentages of patients tested stratified by age, sex, ethnicity, race, and diagnosis of coronary artery disease (CAD). Testing rates were calculated as the number of distinct patients tested per year and as the testing rate per annual patient population. For each stratum we calculated the proportion tested with Wilson 95 % confidence intervals and assessed between-group differences using chi square or Fisher exact tests as appropriate. Annual trends in the testing proportion were modeled using a binomial generalized linear model with a logit link, treating the annual number tested as the numerator and the annual young ischemic stroke population as the denominator, and we report the odds ratio per calendar year with robust standard errors. Geographical variation was visualized using a heat map of testing by state. All analyses were descriptive and intended to characterize population-level patterns of ischemic stroke within the Cosmos network rather than infer causal associations. Given the exploratory design, no additional model-based adjustment for confounding was performed. All data are de-identified in compliance with HIPAA standards and governed under Epic’s “Rules of the Road” for institutional data use.

**Results:**

From 2015 to 2024, out of a total of 188,305 distinct young ischemic stroke patients, 9,226 (4.9 %) underwent Lp(a) testing. Additionally, the annual number of tested patients increased significantly from 179 in 2015 to 1,992 in 2024 (p<0.001), and the annual percentage of patients undergoing Lp(a) testing increased from 4.3 % in 2015 to 9.3 % in 2024. The states with the largest number of tested patients were Ohio (10.4 %), Texas (7.4 %), and Pennsylvania (5.5 %). The rates of testing were significantly different between sexes, with a larger percentage of young women with ischemic strokes tested compared to young men. Analyzing patients with reported racial data, patients who identified as Black or African American underwent testing for Lp(a) at the highest rate, compared with patients who identified as Asian, “None of the above”, White, or Other Race. Among patients undergoing testing with reported ethnic identity, a higher percentage of patients who identified as Hispanic or Latino were tested compared to those who identified as non-Hispanic. Stratifying the total tested patients by age, adults between the ages of 50–60 years made up the largest percentage of patients (4,460; 48.3 %); however, the highest rate of testing occurred in patients aged 5–18. In addition, a higher rate of the young ischemic stroke patients who had ever had a diagnosis of CAD underwent testing compared to patients without CAD.

**Discussion:**

Lp(a) testing among young ischemic stroke patients has increased significantly over the past decade, likely reflecting growing clinical recognition of its causal role in atherosclerotic disease. The rise parallels key updates in lipid management and stroke prevention guidelines, including the 2019 European Society of Cardiology and 2024 National Lipid Association recommendations advocating at least once-in-a-lifetime Lp(a) measurement. Increasing assay availability and heightened awareness of the causal relationship of Lp(a) with atherosclerotic disease may also have contributed to the observed upward trend. Despite this, only about one in twenty young ischemic stroke patients had ever been tested, underscoring a substantial implementation gap between evidence and clinical practice.

The study’s focus on patients aged ≤60 years is particularly relevant given that genetic and structural mechanisms often play a more prominent role in stroke etiology in this younger demographic. In contrast to older adults, where small-vessel disease and atrial fibrillation predominate, younger stroke patients more frequently exhibit monogenic or nontraditional causes, including elevated Lp(a), patent foramen ovale (PFO), and hypercoagulable states.^1–5^ The age threshold of 60 aligns with guideline frameworks for “young stroke” cohorts, which seek to capture patients most likely to benefit from genetic, biomarker-based diagnostic evaluation, and possible PFO closure.^6^ As PFO closure has become increasingly adopted for cryptogenic stroke in younger adults, distinguishing those with an underlying pro-atherogenic risk, such as elevated Lp(a) could further refine treatment selection and secondary prevention strategies (Fig. 1 and Table 1).

Demographic and regional testing differences suggest that local practice norms, clinician specialty focus, and ancestry awareness influence testing behavior. The higher rate of testing among Black and Hispanic patients may partly reflect recognition that these populations often exhibit genetically higher Lp(a) concentrations, predisposing them to premature atherosclerosis^7,8^. Conversely, lower testing in other groups and certain regions may stem from variable institutional protocols or provider awareness. Embedding automated testing prompts in EHR-based stroke order sets could help standardize evaluation across care settings.

Future research should assess how Lp(a) interacts with PFO presence, traditional vascular risk factors, and stroke recurrence to better delineate mechanistic pathways in young patients. Linking Lp(a) levels to outcomes and therapeutic response will be critical to informing personalized prevention and family screening strategies. Despite limitations inherent to EHR-based analysis, these national-scale findings highlight both progress and opportunity in integrating Lp(a) assessment into the modern stroke workup.

## Conclusion

Despite a notable increase in Lp(a) testing in young ischemic stroke patients over the past decade, overall testing rates remain low and inconsistent across demographic and geographic subgroups. These findings highlight a persistent gap between evidence and implementation in a population where genetic and structural causes, such as elevated Lp(a) and patent foramen ovale, often underlie cerebrovascular events. Standardized testing protocols, clinician education, and EHR-based decision support could help close this gap and promote equitable cardiovascular risk identification. As Lp(a)-lowering therapies and precision approaches to PFO closure advance, integrating biomarker screening into the evaluation of stroke patients under 60 years may enable earlier detection of high-risk phenotypes and improve secondary prevention outcomes. Future studies should clarify how Lp(a) influences recurrence and treatment response in this population.

Generative AI and AI-assisted technologies were NOT used in the preparation of this work.

## Supplementary Material

Supplementary material associated with this article can be found, in the online version, at doi:10.1016/j.jstrokecerebrovasdis.2025.108513.

## Figures and Tables

**Fig. 1.**
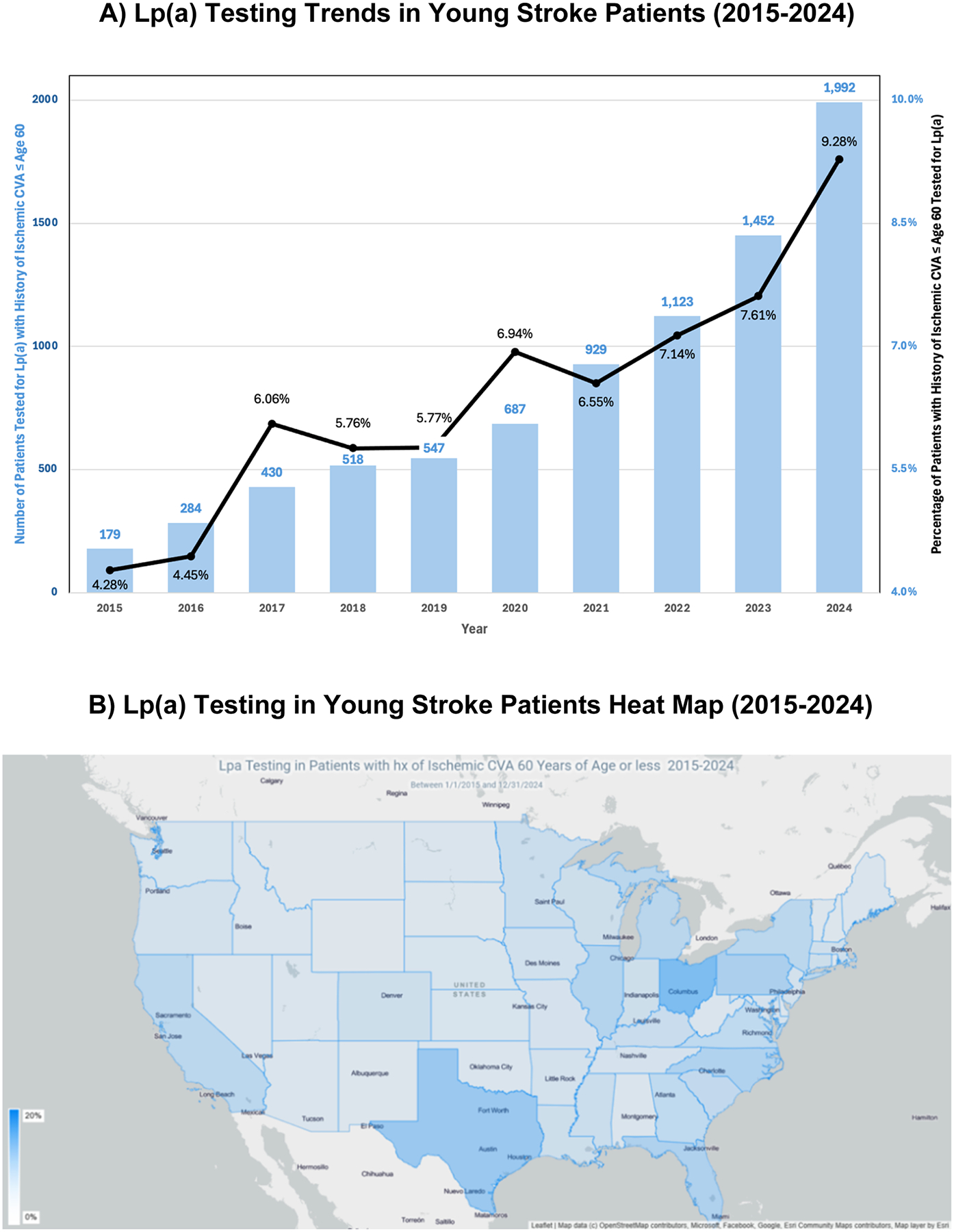
National Trends and Regional Distribution of Lipoprotein(a) Testing in Young Stroke Patients the United States (2015–2024). Panel A: Yearly trends in distinct Lp(a) testing in patients aged 60 or less with history of an ischemic stroke in the United States, showing a substantial increase from 2015 to 2024. Panel B: Geographic distribution of Lp(a) testing in the same population, highlighting regional variation in testing uptake. Created using Epic Cosmos. Analysis performed by Mattheus Ramsis, MD, and internally reviewed by Mattheus Ramsis, MD at UCSD Health on 08/16/25.

**Table 1:** Lp(a) testing proportions with 95 % CIs and overall group comparisons (young ischemic stroke, 2015–2024).

Variable	Category	Tested/Total	% tested (95 % CI)	*P* value
Sex	Male	4,883/102,133	4.78 % (4.65–4.91)	0.018
	Female	4,343/86,148	5.04 % (4.90–5.19)	
	None of the Above	0/24	0.00 % (0.00–13.80)	
Race	White	6,000/122,948	4.88 % (4.76–5.00)	<0.001
	Black or African	2,269/	9.28 % (8.93–9.65)	
	American	24,443		
	Asian	535/6,658	8.04 % (7.41–8.71)	
	Other Race	1,262/48,785	2.59 % (2.45–2.73)	
	None	280/5,317	5.27 % (4.70–5.90)	
Ethnicity	Not Hispanic or Latino	8,099/170,582	4.75 % (4.65–4.85)	<0.001
	Hispanic or Latino	1,127/17,723	6.36 % (6.01–6.73)	
Age	5≤n<18	560/4,933	11.35 % (10.50–12.28)	<0.001
	18≤n<30	606/6,207	9.76 % (9.05–10.53)	
	30≤n<40	1,001/15,891	6.30 % (5.93–6.69)	
	40≤n<50	2,465/43,095	5.72 % (5.50–5.94)	
	50≤n<61	4,460/116,567	3.83 % (3.72–3.94)	
CAD	With	2,181/41,649	5.24 % (5.03–5.45)	<0.001
	Without	7,045/146,656	4.80 % (4.70–4.91)	
